# Supplementary material for: Translational research into the effects of cigarette smoke on inflammatory mediators and epithelial TRPV1 in Crohn’s disease
Source: PLoS One. 2020 Aug 6;15(8):e0236657. doi: 10.1371/journal.pone.0236657 (PMC7410291; doi:10.1371/journal.pone.0236657)

Human

2 x 2 factorial analysis using linear regression, adjusted for age and sex.

ILEUM

TRPV1 protein

Formula: TRPV1_protein = smoking * IBD + age + sex + error

```
Coefficients:
                    Estimate Std. Error t value Pr(>|t|)    
(Intercept)          28.0101     6.3208   4.431 8.41e-05 ***
smokingYes           -3.5211     3.9250  -0.897    0.376    
IBDYes               -5.4920     5.6016  -0.980    0.333    
age                  -0.1835     0.1142  -1.606    0.117    
sexMale              -0.8399     3.2966  -0.255    0.800    
smokingYes:IBDYes     3.0206     7.0970   0.426    0.673    
---
Signif. codes:  0 ‘***’ 0.001 ‘**’ 0.01 ‘*’ 0.05 ‘.’ 0.1 ‘ ’ 1
```

No significant association between TRPV1 protein levels and neither IBD nor smoking.

Post-hoc Tukey multiple comparison testing

IBD.Smoking

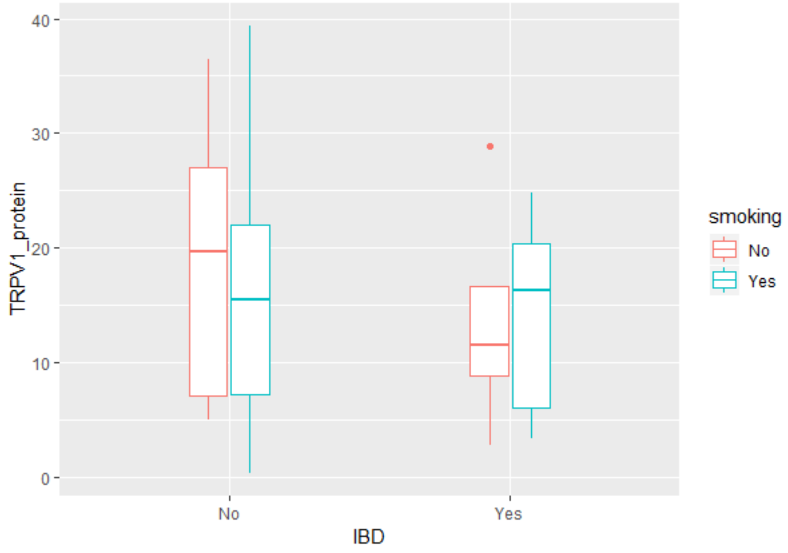

TRPV1 mRNA

Formula: TRPV1_mRNA = smoking * IBD + age + sex + error

```
Coefficients:
                  Estimate Std. Error t value Pr(>|t|)    
(Intercept)       7.595578   0.716067  10.607 6.71e-16 ***
age               0.005272   0.012984   0.406  0.68603    
sexMale          -0.314693   0.389190  -0.809  0.42166    
smokingYes       -0.790906   0.543802  -1.454  0.15057    
IBDYes           -2.440969   0.545572  -4.474 3.10e-05 ***
smokingYes:IBDYes 2.171693   0.767595   2.829  0.00617 ** 
---
Signif. codes:  0 ‘***’ 0.001 ‘**’ 0.01 ‘*’ 0.05 ‘.’ 0.1 ‘ ’ 1
```

IBD is negatively associated with TRPV1 mRNA expression but strength of this association is dependent of smoking status.

Post-hoc Tukey multiple comparison testing

IBD.Smoking

```
Linear Hypotheses:
                      Estimate Std. Error z value Pr(>|z|)    
Yes.No - No.No == 0    -2.4410     0.5456  -4.474   <0.001 ***
No.Yes - No.No == 0    -0.7909     0.5438  -1.454   0.4650    
Yes.Yes - No.No == 0   -1.0602     0.5260  -2.016   0.1818    
No.Yes - Yes.No == 0    1.6501     0.5745   2.872   0.0221 *  
Yes.Yes - Yes.No == 0   1.3808     0.5408   2.553   0.0522 .  
Yes.Yes - No.Yes == 0  -0.2693     0.5568  -0.484   0.9627    
---
Signif. codes:  0 ‘***’ 0.001 ‘**’ 0.01 ‘*’ 0.05 ‘.’ 0.1 ‘ ’ 1
(Adjusted p values reported -- single-step method)
```

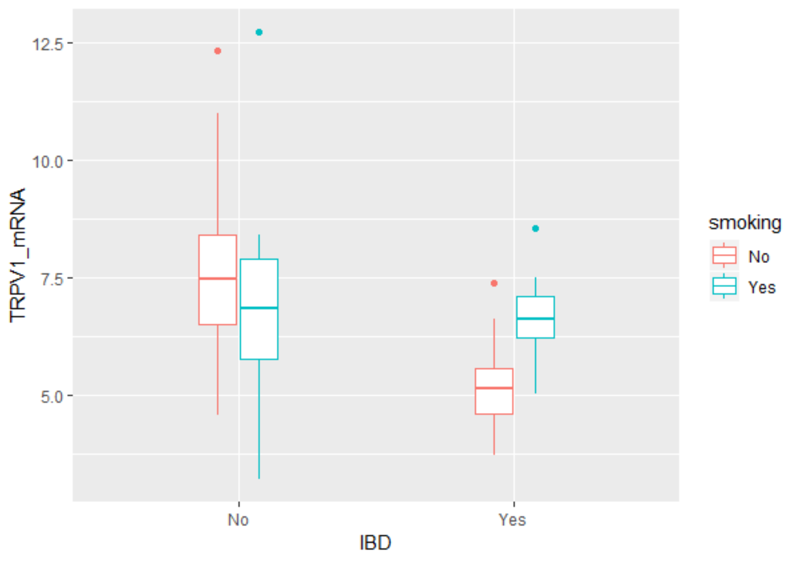

IL-8 mRNA

Formula: IL8 mRNA = smoking * IBD + age + sex + error

```
Coefficients:
                    Estimate Std. Error t value Pr(>|t|)    
(Intercept)         0.956570   0.096656   9.897 1.59e-14 ***
age                 0.002558   0.001776   1.441    0.155    
sexMale            -0.016665   0.053889  -0.309    0.758    
smokingYes          0.913194   0.076611  11.920  < 2e-16 ***
IBDYes              1.444478   0.075095  19.235  < 2e-16 ***
smokingYes:IBDYes  -0.933847   0.106612  -8.759 1.49e-12 ***
---
Signif. codes:  0 '***' 0.001 '**' 0.01 '*' 0.05 '.' 0.1 ' ' 1
```

There is significant association between IBD and smoking on IL-8 mRNA levels. However, this association is influenced by the interaction between IBD and smoking.

Post-hoc Tukey multiple comparison testing

IBD.smoking

```
Linear Hypotheses:
                       Estimate Std. Error z value Pr(>|z|)    
Yes.No - No.No == 0     9.48589    0.80228  11.824  < 1e-07 ***
No.Yes - No.No == 0     4.48591    0.81848   5.481 1.41e-07 ***
Yes.Yes - No.No == 0    9.58168    0.77471  12.368  < 1e-07 ***
No.Yes - Yes.No == 0   -4.99999    0.84832  -5.894  < 1e-07 ***
Yes.Yes - Yes.No == 0   0.09578    0.79020   0.121    0.999    
Yes.Yes - No.Yes == 0   5.09577    0.82411   6.183  < 1e-07 ***
---
Signif. codes:  0 '***' 0.001 '**' 0.01 '*' 0.05 '.' 0.1 ' ' 1
(Adjusted p values reported -- single-step method)
```

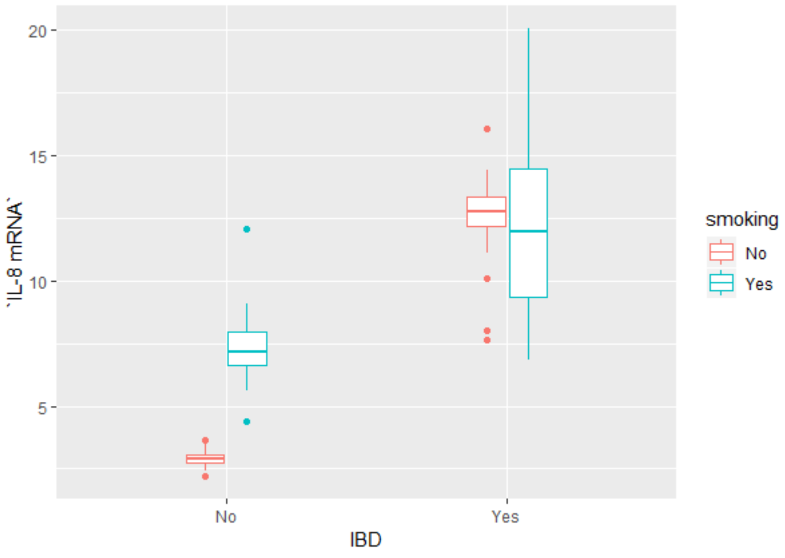

COLON

TPRV1 protein

Formula: TRPV1_protein = smoking * IBD + age + sex + error

```
Coefficients:
                    Estimate Std. Error t value Pr(>|t|)
(Intercept)        18.272327   7.301840   2.502   0.0178 *
age                 0.009833   0.132776   0.074   0.9414
sexMale             4.067300   3.951119   1.029   0.3113
smokingYes         -1.944838   4.469726  -0.435   0.6665
IBDYes             -8.355714   5.622149  -1.486   0.1473
smokingYes:IBDYes  11.950267   7.860122   1.520   0.1386
---
Signif. codes:  0 '***' 0.001 '**' 0.01 '*' 0.05 '.' 0.1 ' ' 1
```

No significant association between TRPV1 protein levels and neither IBD nor smoking.

Post-hoc Tukey multiple comparison testing

IBD.Smoking

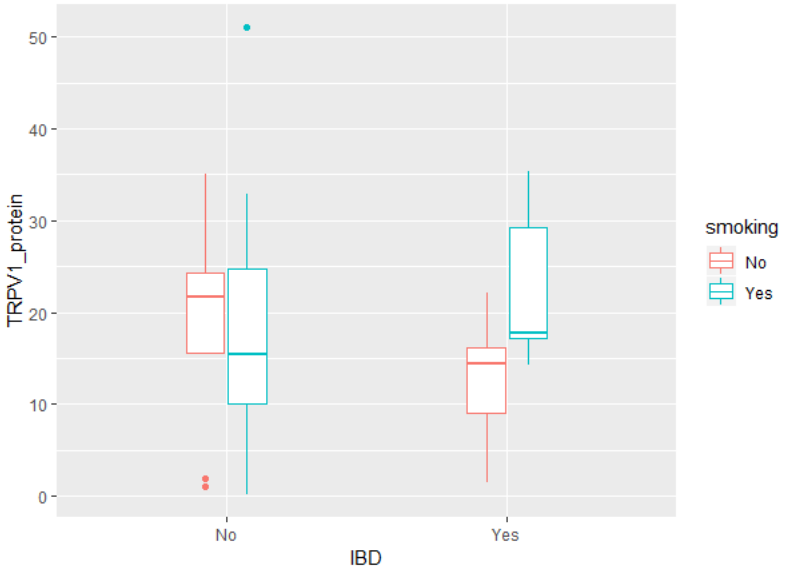

No significant association between smoking and IBD, and TRPV1 protein levels.

TRPV1_mRNA

Formula: TRPV1_mRNA = smoking * IBD + age + sex + error

```
Coefficients:
                   Estimate Std. Error t value Pr(>|t|)    
(Intercept)       2.0519411  0.2115945   9.698 3.64e-13 ***
age              -0.0004104  0.0036491  -0.112  0.91090    
sexMale          -0.0705515  0.1006919  -0.701  0.48670    
smokingYes       -0.3957088  0.1321507  -2.994  0.00423 ** 
IBDYes           -0.2521261  0.1397715  -1.804  0.07716 .  
smokingYes:IBDYes 0.5681486  0.2039298   2.786  0.00747 ** 
---
Signif. codes:  0 '***' 0.001 '**' 0.01 '*' 0.05 '.' 0.1 ' ' 1
```

Post-hoc Tukey test

```
Linear Hypotheses:
                        Estimate Std. Error z value Pr(>|z|)  
Yes.No - No.No == 0      -1.3327     0.9875  -1.350    0.529  
No.Yes - No.No == 0      -2.4265     0.9337  -2.599    0.046 *
Yes.Yes - No.No == 0     -0.0174     1.1274  -0.015    1.000  
No.Yes - Yes.No == 0     -1.0938     1.0095  -1.083    0.698  
Yes.Yes - Yes.No == 0     1.3153     1.0986   1.197    0.627  
Yes.Yes - No.Yes == 0     2.4091     1.1455   2.103    0.151  
---
Signif. codes:  0 '***' 0.001 '**' 0.01 '*' 0.05 '.' 0.1 ' ' 1
(Adjusted p values reported -- single-step method)
```

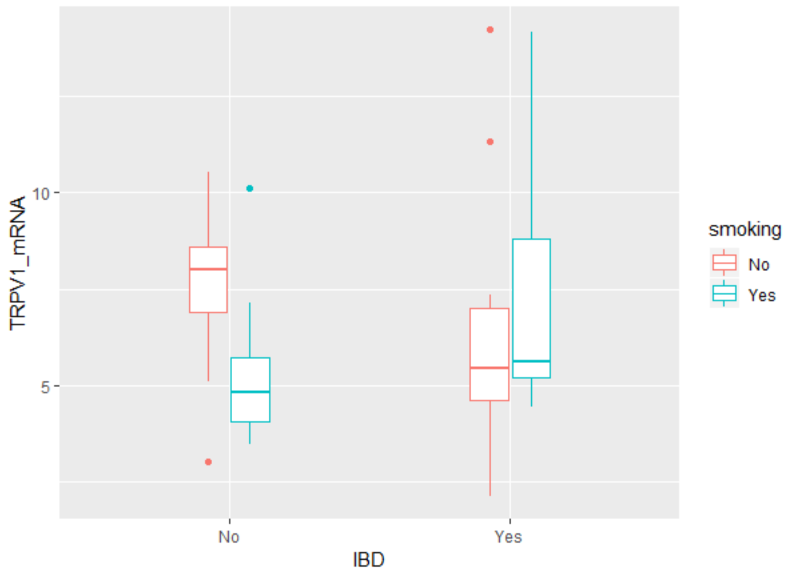

IL-8 mRNA

Formula: IL-8 mRNA = smoking * IBD + age + sex + error

```
Coefficients:
                      Estimate Std. Error t value Pr(>|t|)
(Intercept)           1.424560   0.188466   7.559 2.78e-10 ***
age                   0.008011   0.003348   2.392 0.019884 *
sexMale              -0.084418   0.098255  -0.859 0.393665
smokingYes           -0.042140   0.123803  -0.340 0.734761
IBDYes                0.530283   0.133962   3.958 0.000202 ***
smokingYes:IBDYes     0.245759   0.200612   1.225 0.225346
---
Signif. codes:  0 ‘***’ 0.001 ‘**’ 0.01 ‘*’ 0.05 ‘.’ 0.1 ‘ ’ 1
```

Therefore, IBD generally influences IL-8 levels but smoking does not influence it.

```
Linear Hypotheses:
                          Estimate Std. Error z value Pr(>|z|)
Yes.No - No.No == 0         4.0224     1.0844   3.709  0.00135 **
No.Yes - No.No == 0        -0.5485     1.0021  -0.547  0.94670
Yes.Yes - No.No == 0        5.9262     1.2920   4.587  < 0.001 ***
No.Yes - Yes.No == 0       -4.5709     1.1102  -4.117  < 0.001 ***
Yes.Yes - Yes.No == 0       1.9038     1.2867   1.480  0.44688
Yes.Yes - No.Yes == 0       6.4747     1.3049   4.962  < 0.001 ***
---
Signif. codes:  0 ‘***’ 0.001 ‘**’ 0.01 ‘*’ 0.05 ‘.’ 0.1 ‘ ’ 1
(Adjusted p values reported -- single-step method)
```

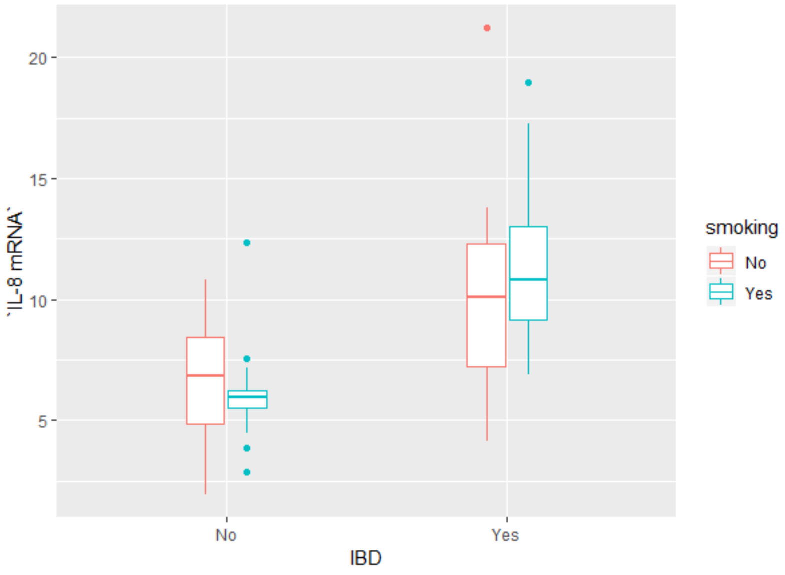

Mouse

Kc mRNA

Formula = Kc mRNA = TNBS + smoking + TNBS*smoking + error

```
Coefficients:
                     Estimate Std. Error t value Pr(>|t|)    
(Intercept)            4.8847     0.7832   6.237 3.77e-07 ***
smokingYes             4.3515     1.1077   3.928 0.000384 ***
TNBSYes                7.2569     1.1077   6.551 1.46e-07 ***
smokingYes:TNBSYes    -1.1835     1.5881  -0.745 0.461126    
---
Signif. codes:  0 '***' 0.001 '**' 0.01 '*' 0.05 '.' 0.1 ' ' 1
```

Smoking and TNBS influence Kc protein but there is no interaction – meaning that smoking does not affect TNBS association

Post-hoc Tukey Test

```
Linear Hypotheses:
                      Estimate Std. Error z value Pr(>|z|)    
Yes.No - No.No == 0      7.257      1.108   6.551   <0.001 ***
No.Yes - No.No == 0      4.351      1.108   3.928   <0.001 ***
Yes.Yes - No.No == 0    10.425      1.138   9.160   <0.001 ***
No.Yes - Yes.No == 0    -2.905      1.108  -2.623   0.0435 *  
Yes.Yes - Yes.No == 0    3.168      1.138   2.784   0.0273 *  
Yes.Yes - No.Yes == 0    6.073      1.138   5.337   <0.001 ***
---
Signif. codes:  0 '***' 0.001 '**' 0.01 '*' 0.05 '.' 0.1 ' ' 1
(Adjusted p values reported -- single-step method)
```

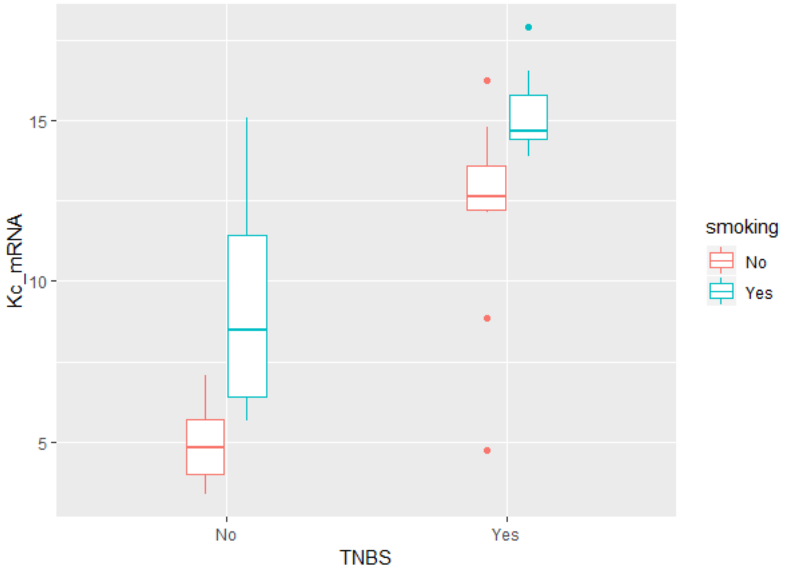

Kc protein

Formula = Kc protein = TNBS + smoking + TNBS*smoking + error

```
Coefficients:
                   Estimate Std. Error t value Pr(>|t|)    
(Intercept)         7.85400    0.26206  29.971   <2e-16 ***
smokingYes         -0.03444    0.36016  -0.096    0.925    
TNBSYes             0.39549    0.37060   1.067    0.295    
smokingYes:TNBSYes  0.75952    0.53847   1.411    0.170    
---
Signif. codes:  0 '***' 0.001 '**' 0.01 '*' 0.05 '.' 0.1 ' ' 1
```

No significant associations found.

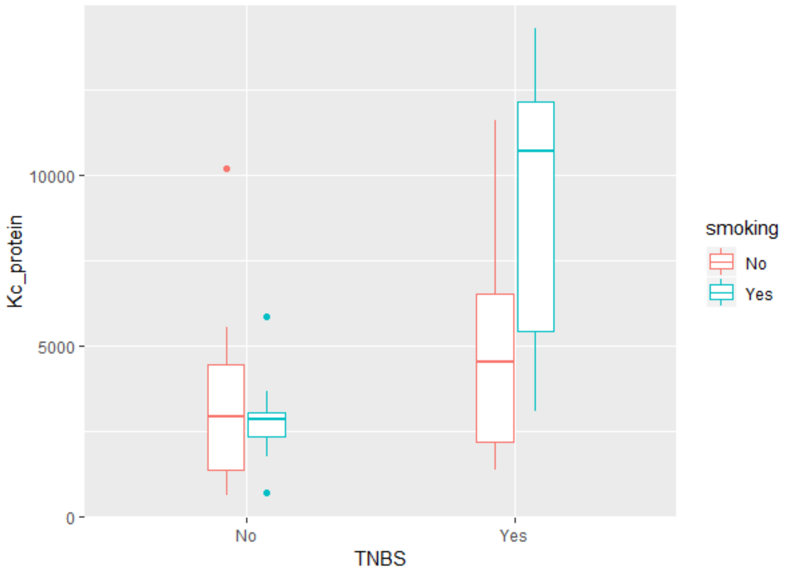

CXCL2 mRNA

Formula = CXCL2 mRNA = TNBS + smoking + TNBS*smoking + error

```
Coefficients:
                  Estimate Std. Error t value Pr(>|t|)    
(Intercept)        12.4118     1.2456   9.964 9.31e-12 ***
smokingYes          3.7019     1.7616   2.101 0.042869 *  
TNBSYes             7.5636     1.7616   4.294 0.000132 ***
smokingYes:TNBSYes  0.4135     2.5256   0.164 0.870906    
---
Signif. codes:  0 ‘***’ 0.001 ‘**’ 0.01 ‘*’ 0.05 ‘.’ 0.1 ‘ ’ 1
```

Smoking and TNBS independently are associated with CXCL2 but there is no interaction between the two (smoking does not affect TNBS activity).

Post-hoc Tukey

```
Linear Hypotheses:
                        Estimate Std. Error z value Pr(>|z|)    
Yes.No - No.No == 0        7.564      1.762   4.294   <0.001 ***
No.Yes - No.No == 0        3.702      1.762   2.101    0.153    
Yes.Yes - No.No == 0      11.679      1.810   6.453   <0.001 ***
No.Yes - Yes.No == 0      -3.862      1.762  -2.192    0.126    
Yes.Yes - Yes.No == 0      4.115      1.810   2.274    0.104    
Yes.Yes - No.Yes == 0      7.977      1.810   4.408   <0.001 ***
---
Signif. codes:  0 ‘***’ 0.001 ‘**’ 0.01 ‘*’ 0.05 ‘.’ 0.1 ‘ ’ 1
(Adjusted p values reported -- single-step method)
```

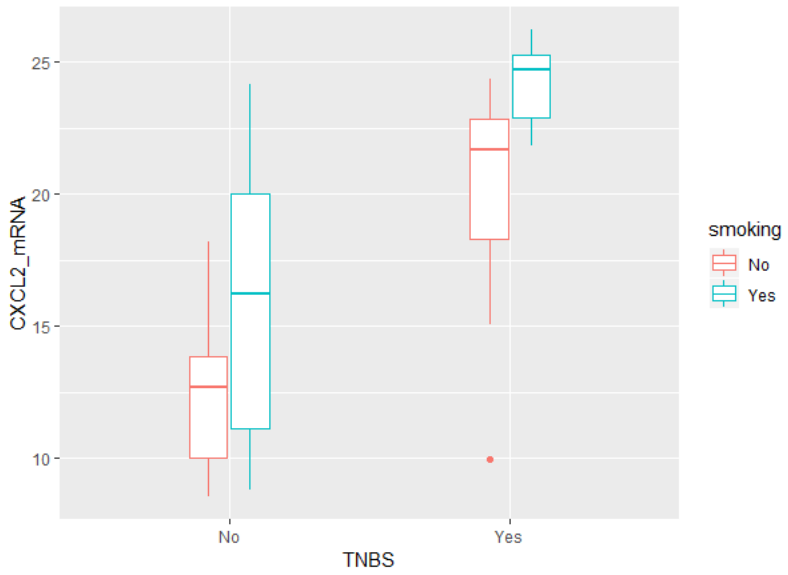

CXCL2 protein

Formula = CXCL2 protein = TNBS + smoking + TNBS*smoking + error

```
Coefficients:
                  Estimate Std. Error t value Pr(>|t|)    
(Intercept)         6.0601     0.4773  12.696 2.12e-12 ***
smokingYes          3.0376     0.6505   4.670 8.77e-05 ***
TNBSYes             1.8913     0.6038   3.132  0.00438 ** 
smokingYes:TNBSYes  0.1776     0.8875   0.200  0.84304    
---
Signif. codes:  0 '***' 0.001 '**' 0.01 '*' 0.05 '.' 0.1 ' ' 1
```

Smoking seems to have an effect on CXCL2 protein.

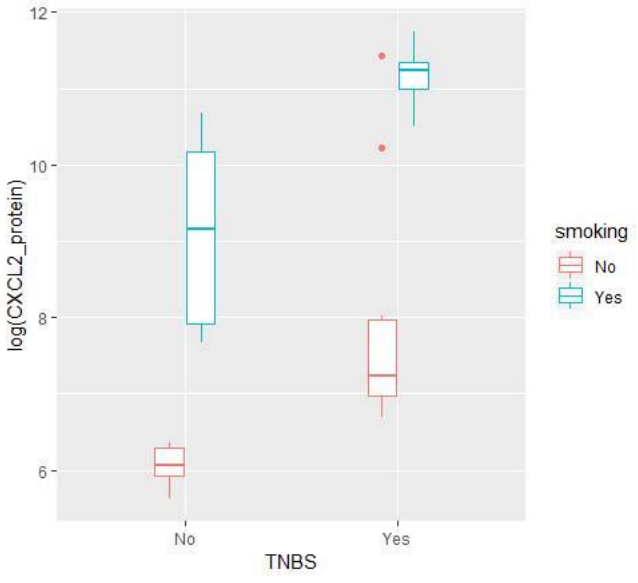

IL-1b mRNA

Formula = IL-1b mRNA = TNBS + smoking + TNBS*smoking + error

```
Coefficients:
                      Estimate Std. Error t value Pr(>|t|)    
(Intercept)            14.2113     0.9105  15.608  < 2e-16 ***
smokingYes              2.2199     1.2876   1.724 0.093528 .  
TNBSYes                 5.3291     1.2876   4.139 0.000209 ***
smokingYes:TNBSYes      0.9366     1.8461   0.507 0.615116    
---
Signif. codes:  0 '***' 0.001 '**' 0.01 '*' 0.05 '.' 0.1 ' ' 1
```

TNBS affects IL-1b but not smoking.

```
Linear Hypotheses:
                        Estimate Std. Error z value Pr(>|z|)    
Yes.No - No.No == 0        5.329      1.288   4.139   <0.001 ***
No.Yes - No.No == 0        2.220      1.288   1.724   0.3110    
Yes.Yes - No.No == 0       8.486      1.323   6.414   <0.001 ***
No.Yes - Yes.No == 0      -3.109      1.288  -2.415   0.0741 .  
Yes.Yes - Yes.No == 0      3.156      1.323   2.386   0.0796 .  
Yes.Yes - No.Yes == 0      6.266      1.323   4.736   <0.001 ***
---
Signif. codes:  0 '***' 0.001 '**' 0.01 '*' 0.05 '.' 0.1 ' ' 1
(Adjusted p values reported -- single-step method)
```

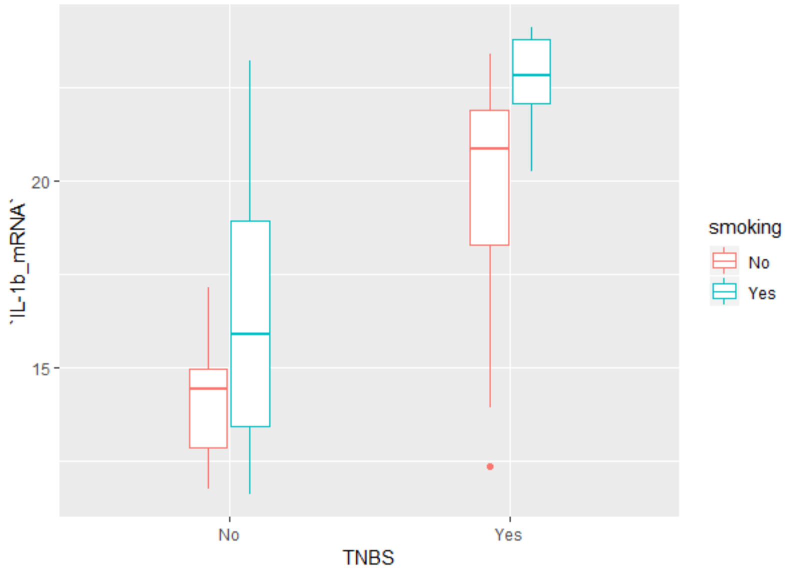

IL-1b protein

Formula = log(IL-1b protein) = TNBS + smoking + TNBS*smoking + error

```
Coefficients:
                      Estimate Std. Error t value Pr(>|t|)    
(Intercept)             3.2802     0.3578   9.167 1.36e-10 ***
smokingYes              1.1335     0.5060   2.240  0.03195 *  
TNBSYes                 1.5015     0.5060   2.967  0.00556 ** 
smokingYes:TNBSYes      0.7804     0.7530   1.036  0.30758    
---
Signif. codes:  0 '***' 0.001 '**' 0.01 '*' 0.05 '.' 0.1 ' ' 1
```

Post-hoc Tukey

```
Linear Hypotheses:
                          Estimate Std. Error z value Pr(>|z|)    
Yes.No - No.No == 0         1.5015     0.5060   2.967  0.01574 *  
No.Yes - No.No == 0         1.1335     0.5060   2.240  0.11202    
Yes.Yes - No.No == 0        3.4154     0.5576   6.125  < 0.001 ***
No.Yes - Yes.No == 0       -0.3680     0.5060  -0.727  0.88597    
Yes.Yes - Yes.No == 0       1.9139     0.5576   3.432  0.00332 ** 
Yes.Yes - No.Yes == 0       2.2819     0.5576   4.092  < 0.001 ***
---
Signif. codes:  0 '***' 0.001 '**' 0.01 '*' 0.05 '.' 0.1 ' ' 1
(Adjusted p values reported -- single-step method)
```

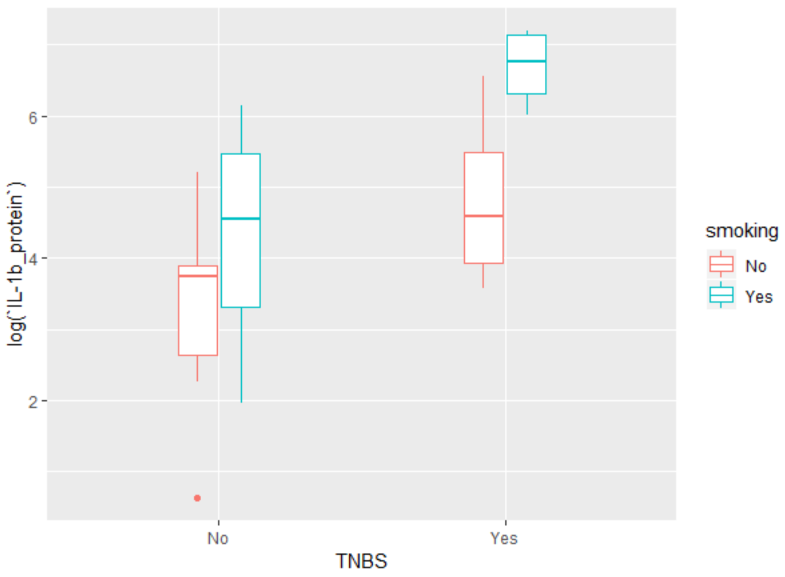

TRPV1 mRNA

Formula = TRPV1 mRNA = TNBS + smoking + TNBS*smoking + error

```
Coefficients:
                      Estimate Std. Error t value Pr(>|t|)
(Intercept)            10.0737     0.2877  35.016   <2e-16 ***
smokingYes              0.5704     0.4069   1.402   0.1700
TNBSYes                 0.6311     0.4180   1.510   0.1404
smokingYes:TNBSYes      1.3369     0.5911   2.262   0.0302 *
---
Signif. codes:  0 '***' 0.001 '**' 0.01 '*' 0.05 '.' 0.1 ' ' 1
```

Significant interaction between smoking and TNBS on TRPV1 mRNA.

```
Linear Hypotheses:
                        Estimate Std. Error z value Pr(>|z|)
Yes.No - No.No == 0      0.63106    0.41800   1.510    0.432
No.Yes - No.No == 0      0.57043    0.40685   1.402    0.498
Yes.Yes - No.No == 0     2.53839    0.41800   6.073   <1e-04 ***
No.Yes - Yes.No == 0    -0.06063    0.41800  -0.145    0.999
Yes.Yes - Yes.No == 0    1.90733    0.42886   4.447   <1e-04 ***
Yes.Yes - No.Yes == 0    1.96797    0.41800   4.708   <1e-04 ***
---
Signif. codes:  0 '***' 0.001 '**' 0.01 '*' 0.05 '.' 0.1 ' ' 1
(Adjusted p values reported -- single-step method)
```

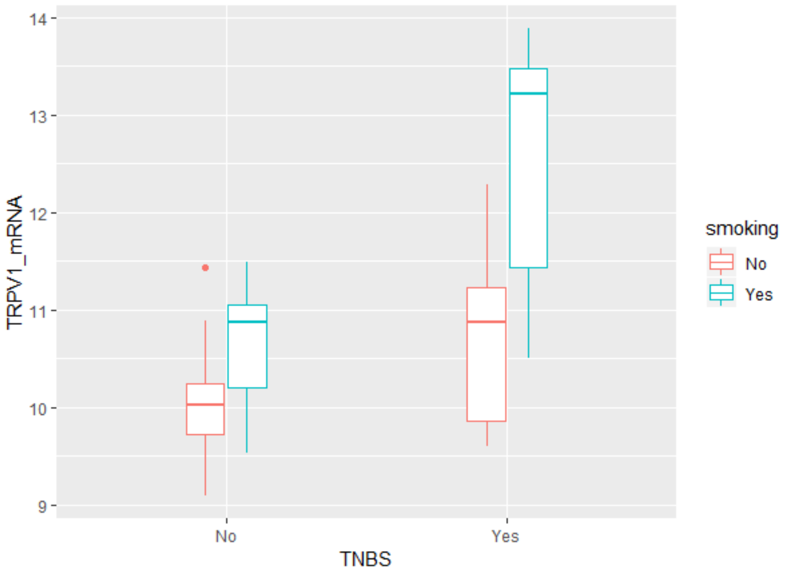

TRPV1 protein

Formula = TRPV1 protein = TNBS + smoking + TNBS*smoking + error

```
Coefficients:
                   Estimate Std. Error t value Pr(>|t|)
(Intercept)          14.199      2.259   6.285  6.3e-07 ***
smokingYes           -2.922      3.195  -0.915    0.368
TNBSYes               1.853      3.389   0.547    0.589
smokingYes:TNBSYes    6.476      5.009   1.293    0.206
---
Signif. codes:  0 ‘***’ 0.001 ‘**’ 0.01 ‘*’ 0.05 ‘.’ 0.1 ‘ ’ 1
```

No significant associations found.

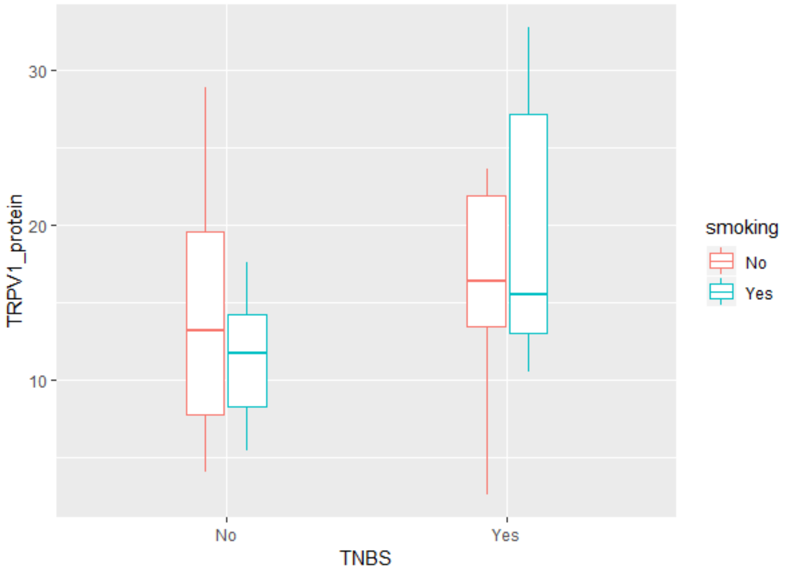

Colon length

Formula = Colon length = smoking + TNBS + smoking*TNBS + error

```
Coefficients:
                    Estimate Std. Error t value Pr(>|t|)    
(Intercept)          8.04000    0.26402  30.452  < 2e-16 ***
smokingYes          -0.15000    0.37338  -0.402  0.69032    
TNBSYes             -1.28000    0.37338  -3.428  0.00157 ** 
smokingYes:TNBSYes -0.07667    0.53532  -0.143  0.88694    
---
Signif. codes:  0 ‘***’ 0.001 ‘**’ 0.01 ‘*’ 0.05 ‘.’ 0.1 ‘ ’ 1
```

TNBS affected colon length but not smoking.

Post hoc Tukey test

```
Linear Hypotheses:
                        Estimate Std. Error z value Pr(>|z|)    
Yes.No - No.No == 0     -1.2800     0.3734  -3.428  0.00337 ** 
No.Yes - No.No == 0     -0.1500     0.3734  -0.402  0.97807    
Yes.Yes - No.No == 0    -1.5067     0.3836  -3.928  < 0.001 ***
No.Yes - Yes.No == 0     1.1300     0.3734   3.026  0.01321 *  
Yes.Yes - Yes.No == 0   -0.2267     0.3836  -0.591  0.93486    
Yes.Yes - No.Yes == 0   -1.3567     0.3836  -3.537  0.00235 ** 
---
Signif. codes:  0 ‘***’ 0.001 ‘**’ 0.01 ‘*’ 0.05 ‘.’ 0.1 ‘ ’ 1
(Adjusted p values reported -- single-step method)
```

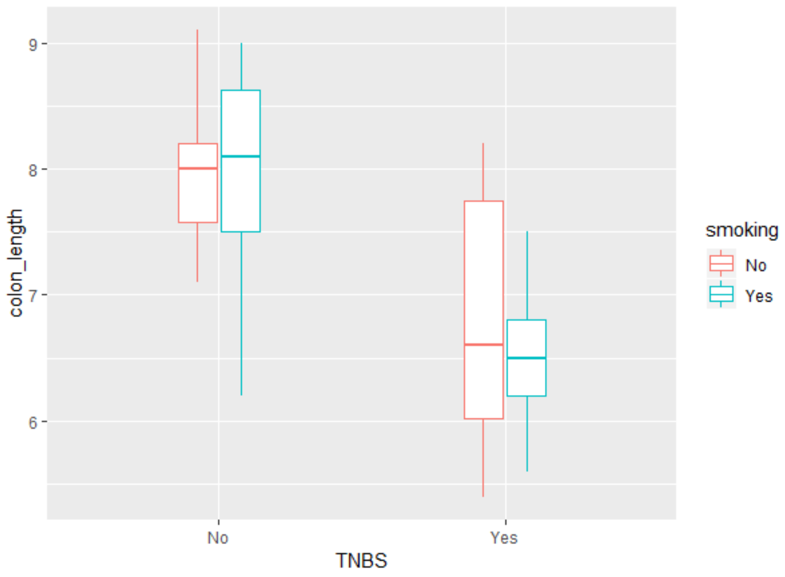

Supplement: S1 File — (PDF) [file pone.0236657.s001.pdf]
