# Supplementary figures and images for: Translational research into the effects of cigarette smoke on inflammatory mediators and epithelial TRPV1 in Crohn’s disease
Source: PLoS One. 2020 Aug 6;15(8):e0236657. doi: 10.1371/journal.pone.0236657 (PMC7410291; doi:10.1371/journal.pone.0236657)

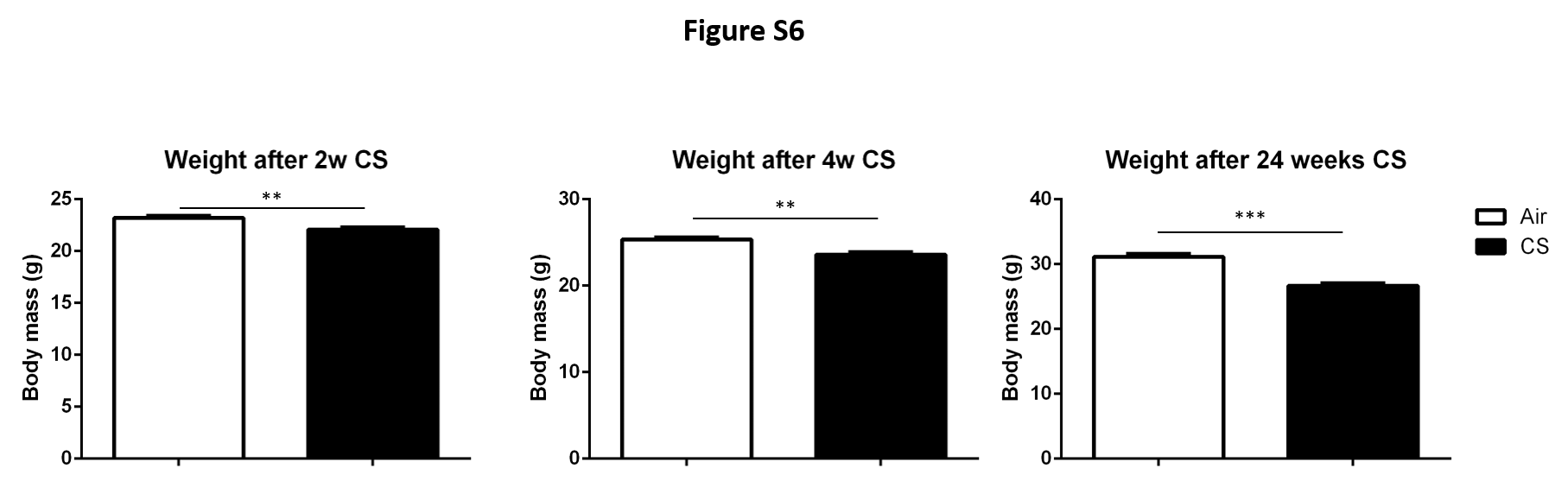

Supplement: S1 Fig — (TIF) [file pone.0236657.s006.tif]
