# Supplementary material for: Translational research into the effects of cigarette smoke on inflammatory mediators and epithelial TRPV1 in Crohn’s disease
Source: PLoS One. 2020 Aug 6;15(8):e0236657. doi: 10.1371/journal.pone.0236657 (PMC7410291; doi:10.1371/journal.pone.0236657)
Supplement: Attachment — Submitted filename: Response to reviewers.docx [file pone.0236657.s007.docx]

Dear Dr. Chamaillard,

We are grateful for the opportunity to submit a revised version of our research article entitled “Translational research into the effects of cigarette smoke on inflammatory mediators and epithelial TRPV1 in Crohn’s disease”, previously submitted as PONE-D-18-00034. We thank the reviewers for their valuable time and useful contribution in the peer review of our work and hereby provide you with a detailed response to the comments raised.

The current manuscript has been approved by all authors and all prevailing local, national and international regulations and conventions, and normal scientific ethical practices, have been respected. No author has an ethical or financial conflict of interest. We affirm that this manuscript is original and has not been published previously nor that is under consideration for publication in another journal. None of the manuscript’s contents has been previously published except in abstract form. We give consent for publication in PloS One, if the manuscript is accepted.

Sincerely yours,

Dr. Liesbeth Allais, on behalf of the authors.

**Journal requirements:**

1. *Please ensure that your manuscript meets PLOS ONE's style requirements, including those for file naming.*

We have adjusted the manuscript to PLOS ONE's style requirements.

2. *Please detail the method of sacrifice in the manuscript.*

The mice were euthanized with an overdose of pentobarbital, which we now clarified in the ‘Materials and Methods’ section. In the paragraph ‘Experimental colitis’, line 164 – 165 now reads as: “The mice were euthanized with an overdose of pentobarbital (Sanofi-Ceva, Paris, France).”

3. *Please include your tables as part of your main manuscript and remove the individual files. Please note that supplementary tables should be uploaded as separate "supporting information" files.*

We have included the tables as part of the main manuscript and removed the individual files. The manuscript was adjusted.

4. *We note that you have indicated that data from this study are available upon request. PLOS only allows data to be available upon request if there are legal or ethical restrictions on sharing data publicly. For more information on unacceptable data access restrictions, please see* [*http://journals.plos.org/plosone/s/data-availability#loc-unacceptable-data-access-restrictions*](http://journals.plos.org/plosone/s/data-availability#loc-unacceptable-data-access-restrictions) *.*

We confirm that we have no legal or ethical restrictions on sharing our data and therefore agree with sharing our data publicly. Our Data Availability statement can be updated accordingly: All relevant data are shown in the paper.

5. *Please include a separate caption for each figure in your manuscript.*

We have included a separate caption for each figure in the manuscript.

**Reviewers' comments:**

**Reviewer 1:**

*In this study, L. Allais et al evaluated the production of neutrophil-specific chemokines and TRPV1 in the intestinal mucosa after exposure to cigarette smoke (CS) and in the context of Crohn’s disease (CD). This study associating the evaluation in patients with or without CD and in murine experimental model includes interesting data elucidating the impact of CS exposure. However, some data should be validated or extended as detailed in my comments. The authors should try to better integrate the data obtained in their different models.*

Major Comments:

1. *It is important to precise the characteristics of the controls in the table 1 or 2. I suppose that they are not healthy controls. Please could you precise the reason explaining the biopsy and if these patients present other diseases? Since there is a crosstalk between the airway and the intestinal mucosa, have the authors controlled the respiratory function in these patients? Is there some histologic lesions in the intestinal biopsies? Is there some difference in the clinical score between smoker and not smoker patients with CD?*

The controls shown in table 1 and 2 are the best option as what can be considered as healthy controls, considering that they voluntarily participated in a screening program for cancer which includes a preventive check-up through colonoscopy. Histological examination confirms the health status, at least of the intestine. In the ‘Materials & Methods’ section, the paragraph ‘Patients’ states at line 124-125: “Control biopsies were obtained from patients who underwent colonoscopy for follow-up of polyp detection or colon carcinoma screening.”

We agree with the author that there is a crosstalk between the airway and the intestinal mucosa and it would be very interesting to elaborate on this crosstalk using the patient cohort in this study. Unfortunately, the respiratory function of the patients has not been controlled in the current study.

We observed typical histological lesions in the intestinal biopsies of CD patients, however, these lesions were not worsened due to smoking. In the healthy controls, no lesions were observed. We have previously shown that cigarette smoke does not cause macroscopical or microscopical damage to the gut (Verschuere et al., 2011 and Allais et al., 2015). Nevertheless, smoking appears to make the small intestine more susceptible to events that trigger inflammation.

2. *The photos reporting the staining with anti-TRPV1 in patients are not convincing. It seems that some binding is present in the mucus layer covering the epithelial layer suggesting that there is some not specific binding. It is important in this case to report a negative control and the expression of TRPV1 in the different groups.*

We agree with the reviewer’s comment and have adjusted figure 1 adding never and currently smoking controls with and without CD (figure 1G and figure 1H). The effect of smoking on TRPV1 mRNA levels is indeed not confirmed by the protein expression levels in our current human study, which indicates a minor effect of smoking on TRPV1 expression in human.

3*. Regarding the expression of chemokines and TRPV1 in mice (fig 2), the analysis was performed at 24 weeks. It might be interesting to analyze their expression at different time points. Interestingly, there is a difference between the figure 2 and the figure 4 for CXCL2 probably related to the different timing. In addition, the authors should validate the differences observed at the mRNA level by the measurement of the chemotactic peptides. Lastly, a photo of TRPV1 staining in air-exposed mice should be reported.*

We have investigated TRPV1 mRNA at 4 and 24 weeks. The results of the 4-week-smoking experiment are shown in Figure 4 and are discussed in the results section ‘Prior cigarette smoke exposure aggravates TNBS-induced pro-inflammatory mediator production and TRPV1 expression in the gut’ (l. 345-360). We agree that extensive temporal evaluation of TRPV1 would have been valuable, but we decided on two time points that can be considered as early and late for mRNA quantification.

The difference in CXCL2 between figure 2 and 4 is, as the reviewer indicates, indeed due to different timing. We have elaborated on the effects of different durations in smoking protocols in Verscheure et al., 2011; Verschuere et al., 2012; Allais et al., 2015; Allais et al., 2016 and Allais et al., 2017. It is indeed interesting to validate the mRNA levels of chemotactic peptides. We have therefore determined the expression of IL-1β, on mRNA and protein level on gut samples of a 4-week-smoking experiment (Figure 4). These findings are described in the results section ‘Prior cigarette smoke exposure aggravates TNBS-induced pro-inflammatory mediator production and TRPV1 expression in the gut’ (l. 345-360).

To conclude, we report a photo of TRPV1 staining in air-exposed mice in Figure 2F.

4. *After treatment with TNBS, it is surprising that there is no difference in the clinical score between air and CS-exposed mice whereas there is a strong effect on chemokines. The authors should evaluate the presence of neutrophils within the mucosa and their activation by i.e . the measurement of MPO. It might be interesting to measure others pro-inflammatory cytokines such as TNF-α As in the first figures, it is essential to report a photo showing the lesions in TNBS- and CS-exposed mice.*

We agree that it would be interesting to evaluate the presence of neutrophils and their activation and will take this into account for future experiments. Regarding pro-inflammatory cytokines, we have previously studied the effect of cigarette smoke exposure on TNF- expression and concluded that inflammation-induction TNF- expression is not worsened by cigarette smoke exposure. These findings were published in Allais et al., 2015 and Allais et al., 2016. As in Allais et al., 2016, we found that cigarette smoke exposure causes an increase of Cxcl2 expression in the ileum. As IL-8, of which Cxcl1 and Cxcl2 are mouse homologues, has been shown to be linked to Crohn’s disease (Kaser et al., 2010), we focused on IL-8 and Cxcl1 and Cxcl2 in this paper. CS in itself does not cause any lesions in the gut, as we have previously reported (Verschuere et al, 2011; Verschuere et al., 2012; Allais et al., 2016). Also, CS could not worsen TNBS-induced lesions, as this is already quite an aggressive inflammatory mouse model. Figure 3D and E show lesions in a PBS- and TNBS-challenged colon respectively.

5. *It seems to have a strong difference between the mRNA (panel A) and the protein expression of TRPV1 in epithelial cell lines particularly for differentiated T84 cells (panel C and D). The expression of TRPV1 seems more important in undifferentiated cells. How the authors differentiated these cells and how they controlled the differentiation of these cells ?*

*This is not specified in the M&M.*
There is indeed a marked difference in *TRVP1* mRNA levels in undifferentiated versus differentiated T84 cells which do not correspond with the observed TRPV1 protein levels. Given the fact that TRPV1 is a transmembrane receptor, the increase in mRNA levels upon T84 differentiation can be attributable to the appearance of a columnar cell architecture with distinct apical and basolateral membrane structures. However, it has been described previously that transcriptional signatures do not necessarily correlate with a comparable functional response, meaning that the presence and level of mRNA transcripts does not guarantee equal protein expression, functionality or cellular location (Devriese *et al.*, 2017). Despite the frequent use of both Caco-2 and T84 cells as models for differentiated intestinal epithelium, the difference in expression of several transmembrane proteins between these two cell lines has also been recognized previously (Bolte *et al.*, 1998, Devriese *et al.*, 2017).

We have now specified the differentiation protocol in l. 174 - 185 of the paragraph ‘Cell culture’ in the Materials and Methods section: “T84 cells were grown in DMEM/nutrient mixture F-12 supplemented with 5% FBS and kept at 37 °C in a humidified atmosphere of air/CO_2_ (95:5, v/v) with three medium changes per week. To induce differentiation, T84 cells were seeded on 24-well, 0.4 μm pore diameter, semipermeable inserts (Greiner Bio-One, Vilvoorde, Belgium) at a density of 10^5^ cells per well and cultured for 3 weeks. Medium was changed three times per week. After this period, the integrity of the monolayer was evaluated by measuring the TEER using a Millicell ERS-2 Voltohmmeter (Merck Millipore, Billerica, MA, USA) to ensure monolayers with TEER values of 700 Ω.cm^2^ or higher were obtained.”

Minor comments
*The table 3 explaining the histologic score is difficult to understand. Please, could you precise what the arrows represent ?*

The downward arrows represent a decreasing effect. The more arrows, the more the effect is decreasing. The upward arrows represent an increasing effect. The more arrows, the more the effect is decreasing.

**Reviewer 2:**

*The authors have investigated in both human Crohn’s disease patients and a mouse model the effects of cigarette smoke on the expression of inflammatory markers in gut tissue. Various effects are presented. I am not competent to judge the appropriateness of the TNBS mouse model or the choice of cytokines/genes investigated.
Data availability: answers regarding data availability are definitely not sufficient, carefully read the requirements and either place your data in a public repository, a supplement or explain the exceptional legal or ethical circumstances which might preclude this.*

Specific Comments:

*Abstract: the abstract should contain more quantitative information, in particular sample sizes (human and mouse) and relevant effect size estimates (with confidence intervals and p-values).*

The human patient and control groups consist of 18-20 subjects/group. In the mouse experiments, each group consisted of 10 mice. We have now clarified the abstract by providing quantitative information. We mentioned all sample sizes and p-values, which are also discussed in the Materials and Methods section and Figure legends. We have also updated the statistical analyses to linear regression for the data on human subjects and the experiments with TNBS colitis mice. The abstract now reads as follows: “Crohn’s disease (CD) is a pathological condition of the gastro-intestinal tract, causing severe transmural inflammation in the ileum and/or colon. Cigarette smoking (CS) is one of the best known environmental risk factors for the development of CD. Nevertheless, very little is known about the effect of prolonged CS exposure on inflammatory modulators in the gut. We examined the effect of CS on cytokine profiles in the healthy and inflamed gut of human subjects and in the trinitrobenzene sulphonic acid (TNBS) mouse model, which mimics distal Crohn-like colitis. In addition, the effect of CS on epithelial expression of Transient Receptor Potential (TRP) channels and their concurrent increase with CS-augmented cytokine production was investigated. Active smoking was associated with increased *IL-8* transcription in ileum of controls (p < 0,001; n = 18-20/group). In the ileum, TRPV1 mRNA levels were decreased in never smoking CD patients (p <0,001; n = 20/group). In the colon, TRPV1 mRNA levels were decreased (p = 0,046) in smoking healthy controls (n = 20/group). Likewise, healthy mice chronically exposed to CS (n = 10/group) showed elevated ileal *Cxcl2* (p = 0,0075) and colonic *Kc* mRNA levels (p = 0,0186), whereas TRPV1 mRNA and protein levels were elevated in the ileum (p = 0,0315). Although CS exposure prior to TNBS administration did not alter disease activity, increased pro-inflammatory cytokine production was observed in the distal colon (Kc: p = 0,0273; Cxcl2: p = 0,104; Il1-β: p = 0,0796), in parallel with the increase of *Trpv1* mRNA (p < 0,001). We infer that CS affects pro-inflammatory cytokine expression in healthy and inflamed gut, and that the simultaneous modulation of TRPV1 may point to a potential involvement of TRPV1 in CS induced production of inflammatory mediators.”

*Page 6, lines 115-123: please give a bit more detail on patient recruitment, were they all recruited at University Hospital Ghent? When? Specific inclusion/exclusion criteria, e.g. regarding duration of illness? Was there any attempt to match on gender (very similar in cases and controls) or to have similar numbers of male and female participants? Was there any overlap between the two control groups (ileum and colon) or were there a total of 78 different control individuals? Any overlap in the cases?*

Patients and controls were recruited at the department of Gastroenterology from the Ghent University Hospital between 2010 and 2015. For smokers, specific inclusion criteria were applied regarding the Pack Year: active smokers had a Pack Year of at least 10. The population was not gender-matched. There is an overlap between the controls and also some patients: 5 patients and 5 controls donated both an ileal and a colonic biopsy. We have adapted the paragraph ‘Patients’ in the Material and Methods section accordingly: “In total, 155 human subjects were examined. All patients were recruited at the Ghent University Hospital between 2010 and 2015. Patients and controls with a Pack Year of at least 10 were included as actively smoking. Ileal biopsies of 19 current smokers and 18 never smokers with CD, and 18 current smokers and 20 never smokers without CD were collected. No selection was made based on gender. In five controls and five patients, ileal and colonic biopsies were taken from the same patient. The characteristics of the ileal biopsy donors are listed in Table 1. Colonic biopsies of 20 never smokers and 20 current smokers with colonic CD, 20 healthy current smokers and 20 healthy never smokers were sampled. The characteristics of the colonic biopsy donors are listed in Table 2. A current smoker was defined as a subject consuming at least 10 cigarettes daily. The CD patients were diagnosed based on the clinical, endoscopic and histological features of Lennard-Jones’ criteria[27]. Control biopsies were obtained from patients who underwent colonoscopy for follow-up of polyp detection or colon carcinoma screening. The controls do not suffer from any inflammatory diseases. The samples were stored in RNAlater at -80°C for qPCR analysis or formalin-fixed, paraffin-embedded and evaluated for histologic inflammation. See Table 1 and 2 for patient characteristics. TRPV1 and IL-8 expression was investigated by immunohistochemistry and qPCR. This study was approved by the local Ethical Committee of University Hospital Ghent (EC UZG 2010/116) and involved Caucasian participants. The above described methods were carried out in accordance with the approved guidelines. Informed consent was obtained from all participating subjects.”

*P 6, L 131 ff: how many animals were there in total and how many in each group?*

Each group consists of 10 mice. Two groups of 10 mice were used in the six-month experiment and four groups of 10 mice were used in the TNBS experiment. In total, 60 mice were sacrificed. We adjusted line 150-152 in the paragraph ‘Animals’ from the ‘Materials and Methods’ section: “Each group consists of 10 mice. Two groups of 10 mice were used in the six-month experiment and four groups 10 mice were used in the TNBS experiment. In total, 60 mice were sacrificed.”

*In the material and methods and also the results part, please make clear which procedures were performed with the human tissue samples and which with mouse samples (e.g. L 173 ff, L 181 ff, L 215ff).*

We have now clarified which procedures were performed on the human tissue samples and which were performed on the mouse tissue samples:

- H&E staining was performed on both human and mouse tissue sections, whereas histological scoring was solely performed on tissue sections from PBS-challenged and TNBS-inflamed mice. Line 198 – 203 in the section ‘H&E staining and scoring’ now reads as: “Paraffin-embedded 4µm tissue sections were taken from ileal and colonic tissue samples, dewaxed and rehydrated. The sections of human and mouse were both stained with H&E. The H&E staining was performed using the Tissue-Tek Prisma/Film automated slide stainer (Sakura, Torrance, US). Only in TNBS-treated mice, the degree of colon inflammation was scored in a blinded manner and independently by two pathologists according to a scoring scheme (Table 3) adapted from Van der Sluis et al., 2006 [31]”.
- Immunohistochemical staining for TRPV1 was performed on both human and mouse tissue sections. Immunofluorescent staining for TRPV1 was only performed on human cell lines. Line 209 – 210 in the section ‘Immunohistochemistry/fluorescence for TRPV1’ now reads as: “Paraffin-embedded sections of human and mouse ileum and colon were dewaxed, after which treatment with Tris/EDTA (pH = 9) was performed for antigen retrieval.”
- Protein extraction followed by Luminex analysis for multiple cytokines was performed on mouse gut samples. The IL-8 ELISA was performed on the supernatant of human cell cultures. Line 245 – 248 in the section ‘Protein extraction, Luminex assay and ELISA’ now reads as: “25-30 µg of snap-frozen mouse distal colon samples were suspended in a 10 times volume of 0.9% NaCl solution with protease inhibitor Complete Roche (Roche, Vilvoorde, Belgium) and pulverized by means of a mechanical TissueLyser LT (Qiagen) at 50 oscillations per second for 2 minutes in pre-chilled eppendorfs.” Line 254 – 255 now reads as: “The IL-8 protein content of human cell culture supernatant was measured by ELISA according to the manufacturer’s instructions (human CXCL8/IL-8 Duoset ELISA, R&D Systems).”

*P11, L 230 ff: the statistical methods and their description are insufficient. In particular, considering the large number of statistical tests that were performed, some kind of adjustment for multiple testing is absolutely necessary. In addition, most of the data come from some sort of two-factorial design (factor one: smoking (yes/no), factor two: in humans case/control, in mice TNBS/PBS treatment; yielding four groups each) which should be considered appropriately in the analysis (e.g. in a linear model with an interaction term) instead of performing simple group comparions (t-test or Mann-Whitney). In most parts of the results it is not really clear which two (of mostly four) groups were compared and which test was used (t-test or Mann-Whitney) for each comparison (e.g. L 248-249: was IL-8 compared between all patients and all controls (smokers and non-smokers together)? L259-260: elevated in smoking CD patients compared to non-smoking CD patients or compared to smoking healthy controls?). Figures would be preferable as box plots instead of bar plots. Finally, an Intraclass Correlation Coefficient analysis is mentioned, but no results from this are presented – however, to evaluate the agreement between histologic scores of two independent pathologists, correlations are inappropriate and an analysis of agreement (e.g.* *Bland-Altman) should be performed.*

We agree with the reviewer’s suggestions. We have now reanalyzed the data using a generalized linear model with smoking, IBD (or TNBS in case of mice) and their interaction as the independent variable, and either expression or protein level as dependent variable. Multiple comparisons within the two-factor linear model was performed, which inherently corrects for multiple testing/false positive detection.

Each figure clearly mentions which groups are compared and which statistical test was used. In Figure 1 and 4 the bar plots were replaced by box plots. We have clarified comparisons and the statistical tests that were used in the results section. Line 262-267 now reads as follows: “Statistical analysis was performed using a general linear model with smoking, IBD status and their interaction as independent variables, and either protein or gene expression level as dependent variable using R version 3.60. Multiple comparison to determine statistically significant pairs was performed using the *multicomp* package.”

We have chosen to depict the groups and their comparisons as bar plots instead of box plots for the clearest visualization of our results. The box plots are provided as supporting information. We agree that the Bland-Altman analysis is a very correct test for comparing two different medical methods. In our study, the Intraclass Correlation Coefficient is used to compare the scoring results of two independent pathological investigators using the same method. We clarified this in the legend of Fig 3F: “Histologic score of inflammation in the distal colon at two days post-TNBS treatment. Colons were excised from anus to caecum. Before measuring, the rectum (5 mm starting from the anus) was removed. The independent investigators show a Cohen’s kappa of 0,287.”

*P 11, L 242: obviously, the mean age of patients was younger than that of controls and the important point is not whether this difference is statistically significant with this sample size but whether age could be a relevant confounder for any of the considered variables (CRP, gene and protein expression). If it could be a confounder, age should be adjusted for in the statistical analysis.*

The mean age of patients is indeed younger than that of controls. We clarified this in the results section, line 271-274 now reads as: “The mean age of the subjects was comparable within the CD and control groups, with a lower median age for CD patients compared to the healthy controls. As each group contains a wide range of ages (shown by IQR in Table 1,2), age was taken into account as a confounding factor.”

*P 11, L 245: for CRP there seems to be a clear pattern of higher values in smokers vs non-smokers (across both cases and controls) and of higher values in cases than controls (across both smoking strata) which might be apparent if appropriately modeled in a two-factorial linear model.*

Given that CRP is an important marker for inflammation, we thought it was interesting to mention our observations concerning of smoking on CRP levels. We agree that this observation deserves thorough investigation. A multi-center study with a larger sample size would be ideal to dig into this matter and provide statistical relevant information on CRP levels in smokers.

*P 13, L291: isn’t colon length shown in fig. 3 C?*

Colon length is indeed shown in Fig 3C. We corrected the sentence, line 351-352 now reads as: “Also, no changes in colon length due CS exposure were observed (Fig 3C).”

*Table 1 and 2: please do not give significances as bars with asterisk in tables, but rather make an additional column to report precise p-values. In table 1 it seems implausible that 1 vs 3 in 5-ASA would be significant while 1 vs 3 in “no medication “ is not; if there are no further missings, the p-value (Fisher’s exact test) should be 0.6 (not significant).*

We have indicated the significances as bars with asterisks in the same way as we indicate significances in the figures. We have performed a Fisher’s exact test with statistical software. We are aware that this is a very small sample size to conclude such findings. We have therefore omitted the analysis of medication use from the manuscript. For a thorough investigation, it would be necessary to set up a multi-center study with a larger sample size to further elaborate on medication use in never versus active smoking CD patients.

*Table 2: only 19 of the 20 smoking control participants took no medication, what kind of medication did this one individual take?*

None of the 20 smoking controls used medication to control IBD symptoms (5-ASA, immunosuppressives, corticosteroids or biologicals). We corrected Table 2 accordingly.
